# Risk Factors for Intestinal and Extraintestinal Cancers in Inflammatory Bowel Disease: A Retrospective Single-Center Cohort Study

**DOI:** 10.3390/cancers17091396

**Published:** 2025-04-22

**Authors:** Rosa Rosania, Maximilian Nord, Florian G. Scurt, Anke Lux, Verena Keitel, Ulrike von Arnim, Marino Venerito

**Affiliations:** 1 Department of Gastroenterology, Hepatology and Infectious Diseases, Otto-von-Guericke University Hospital, 39120 Magdeburg, Germany; maximilian.nord@st.ovgu.de (M.N.); verena.keitel-anselmino@med.ovgu.de (V.K.); ulrike.vonarnim@med.ovgu.de (U.v.A.); m.venerito@med.ovgu.de (M.V.); 2 University Clinic for Nephrology and Hypertension, Diabetology and Endocrinology, Otto-von-Guericke University, 39120 Magdeburg, Germany; florian.scurt@med.ovgu.de; 3 Institute of Biometry and Medical Informatics, Otto-von-Guericke University, 39120 Magdeburg, Germany; anke.lux@med.ovgu.de

**Keywords:** inflammatory bowel disease (IBD), Crohn’s disease (CD), ulcerative colitis (UC), cancers, biologics, immunomodulators

## Abstract

**Background/Objectives**: Patients with inflammatory bowel disease (IBD) face an increased risk of developing intestinal and extraintestinal cancers. This retrospective single-center study aimed to quantify cancer risk and identify potential risk factors associated with cancer in IBD patients. **Methods**: The epidemiological data, disease characteristics, treatment regimens, and occurrences of cancer following IBD diagnosis were collected between January 2021 and February 2022. Hazard ratios (HRs) and standardized incidence ratios (SIRs) were estimated. **Results**: 560 IBD patients were included; 37 patients developed cancer, with 5 patients developing two distinct cancers, resulting in 42 cancers overall. This translated into a twofold increased risk of developing any cancer compared to the general population (SIR 1.94, 95% CI 1.4–2.6). Colorectal (CRC, 29%), skin (19%), and breast cancer (17%) were the most common malignancies. Female patients showed an increased risk for all cancers (SIR 3.1, 95% CI 2.06–4.3), melanoma (SIR 5.6, 95% CI 1.14–16.2), and CRC (SIR 7.5, 95% CI 3–15.4). Conversely, male patients exhibited a significantly increased risk of lymphoma (SIR 26.2, 95% CI 3.2–95.7). Young age at IBD diagnosis and the use of immunomodulators, whether as monotherapy or in combination with biologics, were associated with an increased risk of cancer. **Conclusions**: The risk of CRC and lymphoma in IBD patients may be higher than previously reported, potentially due to the increasing use of combination therapy. Cancer risk in IBD should be regularly assessed and personalized throughout the disease course.

## Simple Summary

Patients with inflammatory bowel disease (IBD) face a higher risk of developing cancer compared to the general population, particularly cancers of the intestine and other organs such as the skin and breast. In this study, we analyzed patient data, including disease type, treatment received, and cancer diagnoses, to better understand who is most at risk. We found that colorectal, skin, and breast cancers were significantly more common among IBD patients. Younger age at IBD diagnosis and the use of immunomodulators, either alone or combined with biologic therapies, were linked to a higher risk of cancer. These findings highlight the importance of tailored cancer screening and monitoring in IBD patients. A better understanding of cancer risk can help doctors make more informed decisions about treatment and long-term care, ultimately improving patient outcomes.

## 1. Introduction

Inflammatory bowel disease (IBD) encompasses a group of chronic, relapsing inflammatory disorders of the gastrointestinal tract, with ulcerative colitis (UC) and Crohn’s disease (CD) being the most prevalent forms [1]. Patients with IBD are at an increased risk of developing both intestinal and extraintestinal cancers compared to the general population [2].

Cancers associated with IBD are commonly categorized into two groups: “inflammation-related cancers”, typically of intestinal origin (with colorectal cancer (CRC) being the most frequent), and “immunomodulator-related cancers”, which are associated with long-term immunosuppressive therapy [2]. In UC, the risk of CRC is further elevated in patients with primary sclerosing cholangitis (PSC), extensive colitis, prolonged disease duration, and a cumulative colonic inflammation affecting over 50% of the colon [2]. However, recent evidence suggests a decline in CRC incidence, likely due to enhanced surveillance and improved management of chronic intestinal inflammation [3]. Immunomodulator-related cancers, such as lymphoproliferative disorders and non-melanoma skin cancer, have been linked to therapies like azathioprine [4,5,6]. Conversely, treatment with 5-Aminosalicylates (5-ASA) has been associated with a reduced CRC risk in UC patients [7]. Moreover, a recent study indicated that combination therapy with 5-ASA and biologics may offer a potential protective effect against cancer in IBD [8]. Despite these findings, comprehensive evaluations of cancer risk in IBD populations remain limited, particularly with respect to differentiating intestinal from extraintestinal malignancies. Variables such as the age at diagnosis, disease extent, and type of treatment vary substantially among IBD patients and may help identify subgroups at increased cancer risk, offering opportunities for personalized care and risk stratification.

The aim of this study is to quantitatively assess the overall cancer risk in IBD patients and to identify specific risk factors for both intestinal and extraintestinal cancers in a cohort referred to a tertiary care outpatient clinic in Germany.

## 2. Materials and Methods

From January 2021 to February 2022, all data of IBD patients attending the IBD outpatient clinic of the University Hospital Magdeburg were retrospectively analyzed. Inclusion criteria were (a) patients with a diagnosis of IBD based on clinical manifestations, endoscopic and imaging features or pathological findings according to the European Crohn’s and Colitis Organization (ECCO) consensus guideline [9]; (b) duration of IBD ≥1 year, including at least two visits to our referral center; (c) no history of cancer prior to IBD diagnosis; (d) detailed clinical features included in the analysis. Patients with a history of cancer prior to IBD diagnosis were excluded from this study.

Clinical data included date of birth, sex, age at study enrolment, age at IBD diagnosis, age at cancer diagnosis, IBD phenotype (CD vs. UC), CD location (L1–L4) CD behavior (B1–B3) and UC extent (E1–E3) according to the Montreal classification [10]. IBD activity was clinically assessed using the partial Mayo score and the Harvey–Bradshaw severity index for patients with UC and CD, respectively. A partial Mayo score between 0 and 1 points was indicative of remission, 2–4 points of mild activity, 5–6 points of moderate activity, and 7–9 points of severe activity in UC patients [11]. A Harvey–Bradshaw severity index <5 points indicates remission, 5 to 7 points of mild activity, between 8 and 16 points moderate, and >16 points severe activity [12].

Patient characteristics and medical history were comprehensively assessed, including smoking status (never, current, or former smoker) and alcohol consumption (never, current, or former drinker). Data were also collected on bowel complications, such as fistulas, stenosis, obstructions, perforations, abdominal abscesses, perianal lesions, and toxic megacolon. Additionally, extraintestinal manifestations (EIMs), including joint, skin, bone, eye, liver, and kidney involvement, as well as associated immune-mediated diseases (asthma, rheumatoid arthritis, psoriasis, multiple sclerosis, autoimmune thyroiditis, and vasculitis), were evaluated [13].

Data were collected on the history of surgery related to IBD, along with details on the type and duration of medical therapies. These included treatment with 5-aminosalicylic acid (5-ASA), glucocorticosteroids, and immunomodulators such as azathioprine (AZA), 6-mercaptopurine (6-MP), and methotrexate (MTX). Biologic therapies, including infliximab (IFX), adalimumab (ADA), golimumab (GLM), vedolizumab (VDZ), and ustekinumab (UST), were assessed both as monotherapy and in combination.

Combination therapy was defined as the concomitant use of an immunomodulator in combination with a biologic.

The diagnosis of cancer was based on pathology. IBD-related cancer was defined as patients who developed cancer within 6 months of IBD diagnosis. For IBD patients with cancer, data were collected on the age at cancer diagnosis, site of primary lesion, type of cancer, duration of IBD, follow-up before cancer diagnosis, and IBD activity before (last recorded visit) and after (first recorded visit) cancer diagnosis.

Continuous variables were expressed as median and interquartile range (IQR), and categorical variables were expressed as percentages. The distribution of demographic characteristics and related factors was compared using the Mann–Whitney U test for continuous data and Fisher’s exact test for categorical variables. A statistical *p*-value of ≤0.05 (two-tailed) was considered significant for all comparisons. To assess the association between risk factors and cancer development, estimated hazard ratios (HRs) in multivariate regression analysis adjusted for age at diagnosis, gender, smoking status, alcohol, and extraintestinal manifestation with corresponding 95% confidence intervals (CIs) were calculated. All statistical analyses were performed using a complete case analysis with the Software SPSS v28 (IBM Corp, Armonk, NY, USA) and IBM SPSS Statistics Essentials for R. Only patients with complete data for all variables included in a specific model or comparison were retained for that analysis.

To determine whether patients with UC or CD have an increased risk of developing cancer compared with the general population, standardized incidence ratios (SIRs) with 95% confidence intervals (CIs) for common and site-specific cancers were calculated in collaboration with the Institute of Biometry and Medical Informatics at the Otto-von-Guericke University Magdeburg. The risk of cancer in the general population was obtained from the annual report of the German Centre for Cancer Registries of the Robert Koch Institute. SIRs were used to compare the observed number of cancers in the IBD study population with the expected number, calculated by sex and age based on national incidence rates from the German Cancer Registry of the Robert Koch Institute. The number of cancer cases that would be expected if patients with IBD had the same risk of cancer as the general population is obtained by multiplying the number of persons–years observed by the incidence rates. The CIs were calculated assuming that the observed number of cases followed a Poisson distribution.

## 3. Results

### 3.1. Baseline Characteristics of IBD Patients

A total of 560 IBD patients (250 UC (127 male/123 female), median age 50 years, range 21–91 years, and 310 CD (130 male/180 female), median age 47 years, range 18–84 years) were included in this study. The age at diagnosis of IBD, IBD duration, median follow-up time, alcohol consumption, and gender did not differ between CD and UC patients.

CD patients were more likely to be current smokers (31%, *p* < 0.001), and UC patients were never smokers (62%, *p* < 0.001). Considering IBD localization according to the Montreal classification [10], the most common site of disease was left-sided disease in UC (E2 stage, 48%, *n* = 121/250) and ileum-colon (L2 stage, 35%, n = 108/310) or ileum (L1 stage, 28%, n = 84/310) in CD.

At the time of data analysis, 56% of UC patients exhibited mild disease activity (*p* < 0.001) and 50% of CD patients were in clinical remission (*p* < 0.001).

EIM were recorded in 33% (187/560) of IBD patients, 32% (101/310) of CD patients, and 34% (86/250) of UC patients. The most common EIMs in UC patients were joints (*p* < 0.002) and skin lesions in CD patients (*p* < 0.01). Surgery related to IBD was performed in 32% (182/560) of IBD patients, most commonly in CD patients (54%, *p* < 0.001).

Demographic and clinical characteristics of the patients are shown in Table 1.

### 3.2. Overall Cancer Occurrence in IBD Patients

Among patients with UC, the median disease duration was 11.5 years (range 5–52 years), while for those with CD, it was 14 years (range 1–53 years).

**Figure 1:**
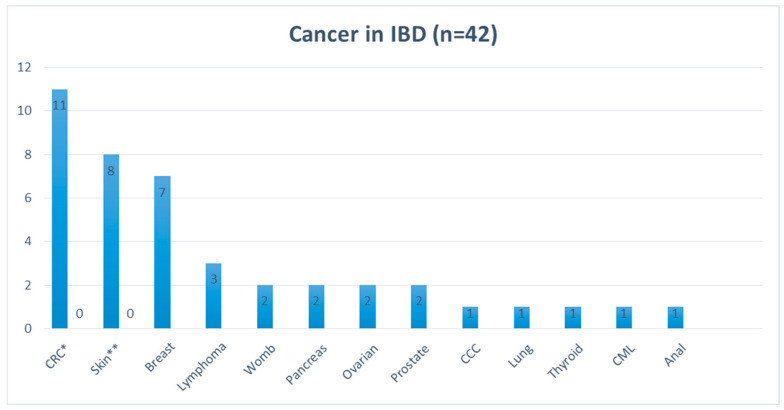
Type of cancer in patients with inflammatory bowel disease. Abbreviations: CRC: colorectal cancer, CCC: cholangiocarcinoma, CML: chronic myeloid leukemia, CRC*: 8 Colon/2 Rectum/1 Sigma, Skin**: 5 Basaliom/3 Melanoma.

During this period, 37 patients (18 UC (7 male, 11 female), median age 50 years, range 30–80 years, and 19 CD (2 male, 17 female), median age 52 years, range 33–82 years) developed cancer. Five patients (2 UC and 3 CD) developed two different types of cancer (42 cancer entities in 37 patients), resulting in a cancer incidence of 6.1% in CD and 7.2% in UC. Of the 42 cancer sites, CRC (29%), skin cancer (19%), and breast cancer (17%) were the most common cancer entities. Figure 1 shows all intestinal and extraintestinal cancers.

At cancer diagnosis, the most common IBD localizations according to the Montreal Classification [10] were left-sided disease (E2 stage, 50%, n = 9/18) and pancolitis (E3 stage, 50%, n = 9/18) in UC and ileum (L1 stage, 37%, n = 7/19) and ileum-colon (L2 stage, 31%, n = 6/19) in CD. Considering the IBD activity at the cancer diagnosis, 59% of CD patients and 50% of UC patients were in clinical remission.

In a subgroup analysis (Table S1), we compared CD patients who developed cancer (19 patients with 22 cancers) to those who did not (n = 291) to better understand the study population and potential differences between these groups. Among CD patients who developed cancer, there was a higher proportion of females compared to males (90% vs. 56%, *p* < 0.06), and they were more likely to have never smoked (100% vs. 67%, *p* < 0.007).

Similarly, we compared UC patients who developed cancer (18 patients with 20 cancers) to those without a history of cancer (n = 232). Detailed information can be found in Table S2.

### 3.3. Immunomodulatory Treatments in IBD Patients

In IBD patients who did not develop cancer, UC patients received a higher proportion of 5-aminosalicylate (93% vs. 43%, *p* < 0.001), glucocorticosteroids (87% vs. 36%, *p* < 0.001), immunomodulators (57% vs. 13%, *p* < 0.001), and anti-TNFα antibodies (56% vs. 22%, *p* < 0.001) compared to Crohn’s disease (CD) patients.

When considering therapy at cancer diagnosis, of 37 IBD patients who developed cancer, 81% (n = 30) were treated with immunomodulators (AZA, 6-MP, or MTX) or biologics (IFX, ADA, GLM, VDZ, or UST) as monotherapy or in combination at the time of cancer diagnosis. Patients with CD were significantly more likely to receive anti-TNFα therapy than patients with UC (58% vs. 11%, *p* < 0.005).

When assessing the duration of any medical treatment before cancer diagnosis, IBD patients (n = 30) were divided into three subgroups as follows:

(1) 15 patients (50%) with therapy duration of less than 5 years (median 2 years, range 0.5–4 years),
(2) 8 patients (27%) with therapy duration between 5 and 10 years (median 7.5 years, range 4–9 years)
(3) 7 patients (23%) with therapy duration exceeding 10 years (median 13.5 years, range 12–21 years).

In CD patients who developed cancer, immunomodulatory treatment was more common compared to those without a history of cancer. Specifically, treatments with immunomodulators (37% vs. 13%, *p* < 0.003), anti-TNFα (58% vs. 22%, *p* < 0.004), and a combination of both (21% vs. 3.7%, *p* < 0.001) were more frequently used. Compared to UC patients without a history of cancer, UC patients who developed cancer were less likely to report treatment with anti-TNFα (56% vs. 11%, *p* < 0.001). However, it is worth noting that among UC patients who developed cancer, only two (11%) were under anti-TNF alpha therapy at the time of cancer diagnosis.

**Figure 2:**
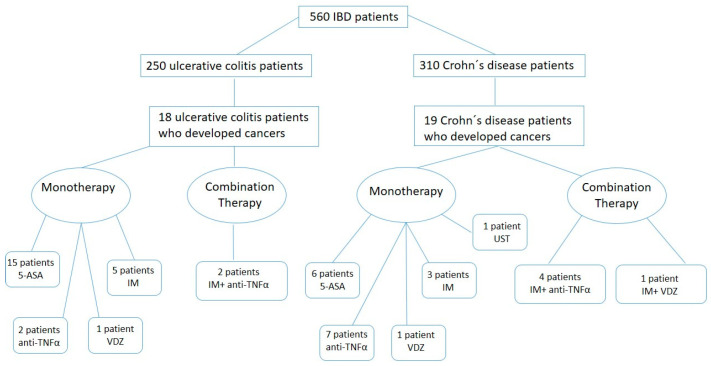
A consort diagram of our study population, detailing the number of patients who developed cancer and their treatment regimens. Combination therapy: the concomitant use of an immunosuppressant (IM) in combination with a biologic. IM immunosuppressants: azathioprine, 6-mercaptopurine, methotrexate; anti-TNFα: infliximab, adalimumab, golimumab. Abbreviations: 5-ASA: 5-Aminosalysilate, VDZ: Vedolizumab, UST: Ustekinumab.

A graphical summary of our study population, detailing the number of patients who developed cancer and their treatment regimens, is shown in Figure 2.

### 3.4. Risk Factors for Cancer Occurrence in IBD Patients

Younger age at first diagnosis of IBD was associated with an increased risk of developing cancer (HR 1.06, 95% CI 1.03–1.09). An inverse association was found in IBD patients with extraintestinal manifestations (HR 0.08, 95% CI 0.01–0.4).

When considering immunomodulatory therapy, combination therapy was associated with an increased risk of cancer (HR 12.77, 95% CI 2.3–70.5). Monotherapy with immunomodulators (HR 188.09, 95% CI 58.7–601.9), anti-TNFα (HR 4.14, 95% CI 1.5–10.8), and UST (HR 3.32, 95% CI 1.1–9.7) similarly exhibited a positive association with cancer risk.

Conversely, a therapy with 5-ASA showed a negative association with cancer risk (HR 0.06, 95% CI 0.02–0.2). Detailed information can be found in Table 2.

### 3.5. SIR

Compared with the general population, IBD patients had a twofold increased risk of developing any type of cancer (SIR 1.93, 95% CI 1.4–2.6).

When considering cancer sites, IBD patients had a fivefold increased risk of developing CRC (SIR 5.07, 95% CI 2.5–9.09) and a twentyfold increased risk of lymphoma (SIR 20.14, 95% CI 4.2–59.6). Detailed information can be found in Table 3.

**Figure 3:**
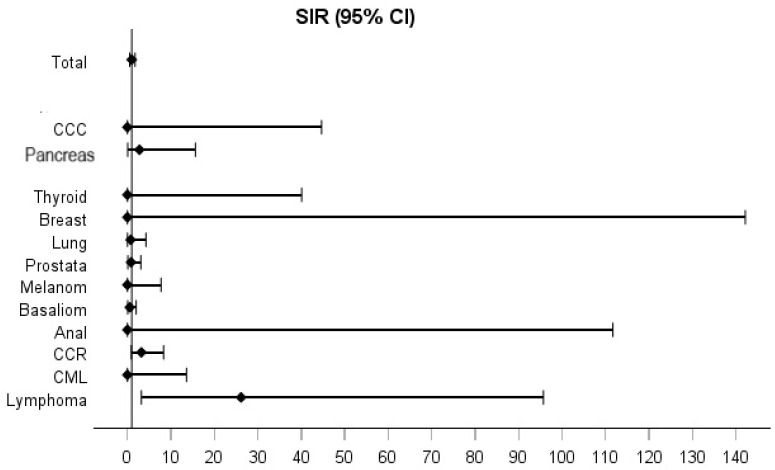
Standardized incidence ratios (SIR) for all intestinal and extra-intestinal malignancies in the male IBD study population.

**Figure 4:**
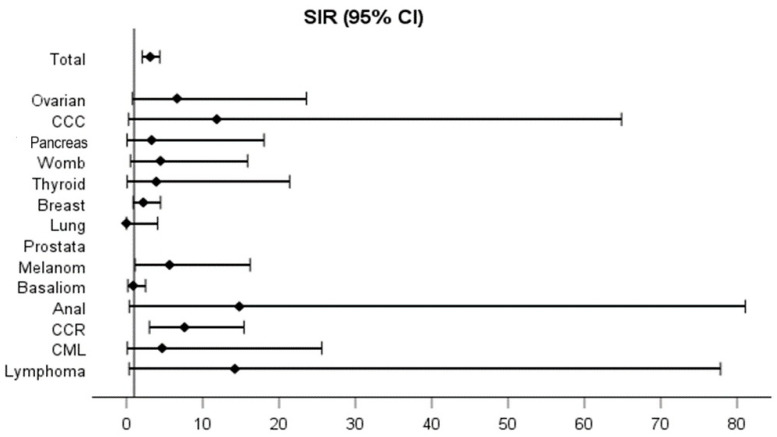
Standardized incidence ratios (SIR) for all intestinal and extra-intestinal malignancies in the female IBD study population.

After stratification by sex, male IBD patients (Figure 3) had a twenty-six-fold increased risk of lymphoma (SIR 26.15, 95% CI 3.2–95.7). On the other hand, female IBD patients (Figure 4) had a threefold increased risk of developing any cancer (SIR 3.1, 95% CI 2.06–4.3), a fivefold increased risk of melanoma (SIR 5.6, 95% CI 1.1–16.2) and a sevenfold increased risk of CRC (SIR 7.59, 95% CI 3–15.4).

## 4. Discussion

According to our data, monotherapy with immunomodulators or biologics, as well as a combination therapy (immunomodulators and biologics), are associated with an increased risk of cancer. In our study cohort, over 80% of IBD patients were being treated with biologics and/or immunomodulators at the time of their cancer diagnosis. Additionally, 50% of these patients had been on this therapy for less than 5 years. This suggests that patients with severe IBD requiring more intensive treatment may face an increased risk of developing cancer.

Similar to previous studies [14,15], we observed a twofold increased risk of developing any type of cancer in IBD patients compared to the general population. However, the twenty-fold increased risk of lymphoma in our study is notably higher than that previously reported in the literature. A recent Scandinavian cohort study found a 17.1-fold increased risk of lymphoma in CD patients and a 14.3-fold increased risk in UC patients [16]. The Scandinavian study also reported an increased lymphoma risk in IBD patients exposed to immunomodulators (HR 1.8 in UC, 2.3 in CD) and combination therapy (HR 2.5 in CD, 3.4 in UC) [16]. Notably, this study did not specify the types of biologics used in combination therapy or differentiate between first- and second-line biologic treatments, which may impact risk estimates. Over the past two decades, the expanding range of IBD treatments—including immunomodulators, biologics, and combination therapy—may contribute to variations in reported lymphoma risk across studies. A recent meta-analysis reported that the increased risk of lymphoma was observed only in IBD patients with at least 1 year of current thiopurine exposure (SIR: 5.71), and this risk returned to baseline levels after the discontinuation of thiopurines. This suggests that a short induction of combination therapy might minimize long-term cancer risk. Randomized clinical trials are needed to confirm the data.

While our findings support the overall trend of elevated lymphoma risk in IBD, the higher incidence observed in our cohort may reflect population differences, treatment regimens, or methodological approaches.

Additionally, in line with the Scandinavian study [16], we confirmed that male IBD patients have an even higher risk of developing lymphoma, with a 26-fold increased risk after stratification by gender. This finding is further supported by a French nationwide cohort study [17], which demonstrated a heightened lymphoma risk in IBD patients receiving thiopurine monotherapy (HR 2.6), anti-TNF monotherapy (HR 2.4), or combination therapy (HR 6.1) compared to unexposed IBD patients. These significant differences highlight the need to explore underlying factors, such as population characteristics, treatment exposures, and duration.

Regarding overall cancer risk, a French population-based study from 2023 on pediatric-onset IBD [17] reported an HR from 6.1 to 7.4 in patients under therapy with anti-TNFα and combination therapy. To our knowledge, our adult IBD cohort shows the highest HR for both intestinal and extraintestinal cancers under combination therapy. In multivariate analysis, we observed increased HRs in IBD patients treated with combination therapy or monotherapy with immunomodulators, anti-TNFα, or UST. Notably, the HR for immunomodulator monotherapy was particularly high (HR 188.09, 95% CI 7–601.9), but the wide confidence interval suggests potential confounding and a small number of events. Therefore, these findings should be interpreted with caution due to the retrospective study design. Interestingly, VDZ therapy was not associated with an increased cancer risk in our analysis. Environmental factors such as occupational exposure, diet, and others (see Table 2) likely play an important role in cancer development. However, due to the retrospective nature of our study, we lacked complete data on these variables. This represents an important limitation, and prospective studies integrating environmental risk factors are needed. Our study also lacked the statistical power to assign increased risk to specific cancer subtypes, and being a tertiary care center, we may have captured a population with more severe disease, which could partly explain the high cancer rates. However, the risk of developing cancer in our cohort was similar to that observed in other studies.

In our study, CRC was the most common cancer. The five-fold increased risk of developing CRC in our IBD patients compared to the general population was much higher than previously observed. Indeed, a meta-analysis of population-based studies up to 2009 described a SIR of 1.7 for CRC in IBD patients compared to the general population [18]. A more recent study from Northern California conducted between 1998 and 2010 found SIRs for CRC in IBD patients of 1.6 [19]. The most recent (2017–2020) population-based study from Ontario reported an SIR of 1.7 [20] for CRC in IBD patients compared to the general population. Conversely, a Danish nationwide study from 1999 to 2008 even reported a lower CRC risk (SIR: 0.5) [21]. Among our CRC cases (n = 11), over 50% were treated with immunomodulators and/or biologics, and 73% had clinical moderate to severe disease activity at the time of their cancer diagnosis. This suggests that the combination of local chronic inflammation and immunomodulatory therapy and/or biologic therapy may be critical contributors to CRC risk.

In line with previous studies, our multivariate regression model confirmed that combination therapy, immunomodulators, and anti-TNFα were associated with increased cancer risk [22,23,24,25,26]. Moreover, 50% of our IBD patients who developed cancer had been treated with these therapies for a median of 2 years (range 0.5–4 years).

Our study findings, in contrast, suggest that treatment with 5-ASA is inversely associated with the risk of cancer. These results align with a recently published study [7] indicating a potential chemopreventive effect of 5-ASA in reducing the risk of colorectal cancer (CRC) in patients with inflammatory bowel disease (IBD). The protective effect of 5-ASA may be attributed to its anti-inflammatory properties, which help suppress chronic inflammation—a key driver of CRC development in IBD. Additionally, 5-ASA has been shown to modulate immune responses, reduce oxidative stress, and inhibit tumor-promoting pathways, all of which may contribute to its role in lowering CRC risk [27].

A key limitation of our study is its retrospective, single-center design, which may introduce selection bias and limit generalizability. The small sample size also reduces statistical power. Although a monocentric approach ensures standardized data collection, it may not reflect broader patient populations. We also lacked detailed patient characteristics, which limits the interpretation of observed sex-based differences. Variables like lifestyle, hormones, genetics, and cumulative medication exposure could all be relevant, but our dataset did not allow for in-depth analysis. To strengthen these findings, future multicenter or population-based studies with larger cohorts and more comprehensive data are needed.

## 5. Conclusions

In conclusion, our study highlights an increased risk of both lymphoma and CRC in IBD patients, particularly among those treated with immunomodulatory therapy—whether as monotherapy or in combination with anti-TNFα agents.

Given the elevated cancer risk, individualized risk assessment is essential at every stage of IBD management, taking into account the type and duration of immunomodulatory therapy (whether used alone or in combination) as well as long-term chronic inflammation. Our findings suggest that a limited course of combination therapy might reduce long-term cancer risk, but this hypothesis requires validation in prospective, randomized clinical trials.

## Figures and Tables

**Table 1:** Baseline characteristics of our study population.

	IBD (560)	CD (310)	UC (250)	*p*
Age at IBD diagnosis, median [range], y	31 (6–76)	30 (6–70)	32 (5–76)	0.1
IBD duration **, median [range], y	14 (1–53)	14 (1–53)	15 (5–52)	0.1
Median follow-up ^, median [range], y	10 (1–26)	9 (1–23)	10 (4–26)	0.1
Gender, No. (%)				
females	303 (54%)	180 (58%)	123 (49%)	0.03
males	257 (46%)	130 (42%)	127 (51%)	
Smoking habits (%)				
current	116 (21%)	97 (31%)	19 (8%)	<0.001 *
never	279 (50%)	124 (40%)	155 (62%)	<0.001 *
former smoker	41 (7%)	16 (5%)	25 (10%)	0.03 *
not specified	124 (22%)	73 (24%)	51 (20%)	0.3
Alcohol intake (%)				
current	26 (5%)	17 (5%)	9 (4%)	0.2
never	389 (69%)	206 (67%)	183 (73%)	0.08
former	6 (1%)	4 (1%)	2 (1)	0.5
not specified	139 (25%)	83 (27%)	56 (22%)	0.2
Montreal Classification				
		^a^ L1 84 (28%)	^b^ E1 34(13%)	
		L2 108 (35%)	E2 121 (48%)	
		L3 79 (25%)	E3 87 (35%)	
		L4 39 (12%)	-	
		^c^ B1 169 (55%)	-	
		B2 71 (23%)		
		B3/p 70 (22)	-	
Disease activity				
*remission*		^d^ 155 (50%)	^e^ 60 (24%)	<0.001 *
*mild*		73 (23%)	140 (56%)	<0.001 *
*moderate*		77 (25%)	50 (20%)	0.1
*severe*		5 (2%)	0	
Extra-intestinal manifestations	187 (33%)	101 (32%)	86 (34%)	0.6
*joints lesions*	88 (47%)	36 (36%)	52 (60%)	0.002 *
*skin lesions*	58 (31%)	41 (40%)	17 (20%)	0.01 *
*eyes lesions*	14 (7%)	7 (7%)	7 (8%)	0.6
*kidneys lesions*	5 (3%)	5 (5%)	0	
*liver lesions*	22 (12%)	12 (12%)	10 (12%)	0.9
IBD-related surgery, No. (%)	182 (32%)	167 (54%)	15 (6%)	<0.001 *

Continuous variables are expressed as median and range (IQR). Categorical variables are expressed as percentages. Abbreviations: IBD: inflammatory bowel disease; CD: Crohn’s disease; UC: ulcerative colitis. Montreal classification: ^a^ Localization of CD: L1: ileitis; L2: colitis; L3: ileo-colitis; L4: upper gastrointestinal tract. ^b^ Localization of UC: E1: proctitis, E2: left-sided colitis, E3: pancolitis. ^c^ Behaviors of CD: B1: inflammatory, B2: structuring, B3: penetrating. Disease activity in data analysis was determined using the ^d^ Harvey–Bradshaw Severity Index for CD and ^e^ MAYO score for UC. * *p* < 0.05, ** IBD duration from IBD first diagnosis to data analysis (2021), ^ median follow-up from first visit at our IBD outpatient clinic to data analysis (2021).

**Table 2:** Risk factors for cancer occurrence in IBD patients.

Variables	HR_univariate_ (95% CI)	HR_multivariate_ (95% CI)
Age	1.07 (1.04–1.09) ^a^	1.06 (1.03–1.09) ^a^
Female sex	1.42 (0.7–2.8)	1.65 (0.7–3.8)
Smoking habits		
Never	1.0 (ref.)	1.0 (ref.)
Yes	1.71 (0.6–4.2)	1.41 (0.2–9.4)
In the past	1.89 (0.6–5.4)	2.30 (0.7–7.5)
Alcohol intake		
Never	1.0 (ref.)	1.0 (ref.)
Yes	1.39 (0.1–10.3)	1.35 (0.1–15.3)
In the past	NA ^b^	NA
Extraintestinal manifestations	0.22 (0.07–0.7) ^a^	0.08 (0.01–0.4) ^a^
Medication		
Combination therapy vs. monotherapy ^c^	4.73 (2.001–11.2) ^a^	12.77 (2.3–70.5) ^a^
Monotherapy		
5-Aminosalysilate	0.58 (0.3–1.1)	-
Steroid	0.01 (0.001–0.1) ^a^	-
IM ^d^	8.42 (4.04–17.5) ^a^	-
anti-TNFα ^e^	1.12 (0.5–2.2)	-
Vedolizumab	1.14 (0.2–4.8)	-
Ustekinumab	3.32 (1.1–9.7) ^a^	-
Combination therapy ^c^		
IM + anti-TNFα ^e^	10.07 (4.1–24.6) ^a^	-
IM + Vedolizumab	1.56 (0.2–11.5)	-
IM + Ustekinumab	0.04 (0.001–2496.4)	-

HR: Hazard Ratio. ^a^ adjusted for Age at diagnosis, gender, smoking status, alcohol, and extraintestinal manifestation. ^b^ *p* < 0.05, ^c^ Combination therapy: the concomitant use of an immunosuppressant (IM) in combination with a biologic. ^d^ IM immunosuppressants: azathioprine, 6-mercaptopurine, methotrexate; ^e^ anti-TNFα: infliximab, adalimumab, golimumab. NA: Not applicable.

**Table 3:** Standardized incidence ratios (SIR) for all intestinal and extra-intestinal malignancies in the IBD study population. Abbreviations: CML: chronic myeloid leukemia, CRC: colorectal cancer, CCC: cholangiocarcinoma.

Cancers (Total)	Observed Cases	Expected Case/Year	Expected Case/Follow Up	SIR	95% CI
Lymphoma	3	0.016	0.147	20.41	4.2–59.6
CML	1	0.053	0.489	2.040	0.05–11.4
CRC	11	0.233	2.167	5.07	2.5–9.09
Anus	1	0.011	0.101	9.89	0.2–54.7
Basalioma	5	0.762	7.087	0.70	0.2–1.6
Melanoma	3	0.109	1.015	2.95	0.6–8.6
Prostate	2	0.255	2.369	0.84	0.1–3.09
Lung	1	0.238	2.209	0.45	0.01–2.5
Breast	7	0.344	3.199	2.18	0.8–4.4
Thyroid	1	0.038	0.350	2.86	0.07–15.8
Uterus	2	0.048	0.448	4.46	0.5–15.8
Pancreas	2	0.072	0.665	3.00	0.3–10.8
CCC	1	0.018	0.168	5.94	0.1–33.07
Ovary	2	0.032	0.302	6.63	0.7–23.5
Total	42	2.33	21.68	1.937	1.4–2.6

## Data Availability

No data is unavailable due to privacy or ethical restrictions.

## Supplementary Materials

The following supporting information can be downloaded at https://www.mdpi.com/article/10.3390/cancers17091396/s1, Table S1: Comparison of the demographic and medical data of Crohn’s disease patients who developed cancer (19 patients with 22 cancers) with Crohn’s disease patients who did not develop cancer (n = 291). * *p* < 0.05; Table S2: Comparison of the demographic and medical data of ulcerative colitis patients who developed cancer (18 patients with 20 cancers) with ulcerative colitis patients who did not develop cancer (n = 232). * *p* < 0.05.

## Abbreviations

IBD	Inflammatory bowel disease
UC	Ulcerative colitis
CD	Crohn’s disease
CRC	Colorectal cancer
PSC	Primary sclerosing cholangitis
5-ASA	5-Aminosalicylates
ECCO	European Crohn’s and Colitis Organization
AZA	Azathioprine
6-MP	6-mercaptopurine
MTX	Methotrexate
anti-TNFα	Infliximab, Adalimumab, Golimumab
IFX	Infliximab
ADA	Adalimumab
GLM	Golimumab
VDZ	Vedolizumab
UST	Ustekinumab
IQR	Interquartile range
HR	Hazard ratio
CI	Confidence interval
