# Supplementary material for: Risk Factors for Intestinal and Extraintestinal Cancers in Inflammatory Bowel Disease: A Retrospective Single-Center Cohort Study
Source: Cancers (Basel). 2025 Apr 22;17(9):1396. doi: 10.3390/cancers17091396 (PMC12071157; doi:10.3390/cancers17091396)
Supplement: Supplementary file 1 [file cancers-17-01396-s001.zip › cancers-3520636-supplementary.pdf]

Supplementary Materials

Table S1. Comparison of the demographic and medical data of Crohn´s disease patients who developed cancer (19 patients with 22 cancers) with Crohn´s disease patients who did not develop cancer (n = 291). * $p < 0.05$.

	**CD (291)**	**CD with cancer (22/19)**	**p**
Age at diagnosis of IBD, median [range], y	30 (6-70)	36 (15-75)	0.66
IBD duration, median [range], y	14. (1-53)	16 (1-35)	0.38
Sex, No. (%)			
females	163 (56%)	17 (90%)	**0 .006***
males	128 (42%)	2 (10%)	
Smoking habits (%)			
current	93 (31%)	4 (21%)	0.34
never	112 (39%)	12 (64%)	0.04
former smoker	15 (5%)	1 (5%)	0.98
not specified	71 (24%)	2 (10%)	0.22
Alcohol intake (%)			
current	17 (5%)	0	
never	206 (67%)	19 (100%)	**0.007***
former	4 (1%)	0	
not specified	83 (27%)	0	
Montreal Classification			
	[a]L1 77 (26%)	[a]L1 7 (37%)	0.35
	L2 102 (35%)	L2 6 (31%)	0.7
	L3 76 (25%)	L3 3 (16%)	0.3
	L4 36 (12%)	L4 3 (16)	0.6
	[c]B1 159 (55%)	[c]B1 10 (53%)	0.9
	B2 68 (23%)	B2 3 (16%)	0.4
	B3/p 64 (22)	B3/p 6 (31%)	0.3

Disease activity			
remission	[d]144 (50%)	[d] 11 (59%)	0.5
mild	67 (23%)	6 (31%)	0.4
moderate	75 (25%)	2 (10%)	0.15
severe	5 (2%)	0	
Extra-intestinal manifestations	101 (32%)	0	
IBD-related surgery, No. (%)	157 (54%)	10 (52%)	0.91
Medication	MC (n=291)		
5- Aminosalicylate	126 (43%)	6 (31%)	0.31
Glucocorticoids	104 (36%)	0	
IM (AZA/6-MP/MTX)	37 (13%)	3 (16%)	**0.003***
anti-TNFα	64 (22%)	7 (37%)	**0.004***
Vedolizumab	19 (6%)	1 (5%)	0.82
Ustekinumab	32 (11%)	1 (5%)	0.4
Combination therapy			
IM+anti-TNFα	11 (3.7%)	4 (21%)	**0.0006***
IM+ Ustekinumab	13 (4.4%)	0	
IM+ Vedolizumab	5 (1.7%)	1 (5%)	0.27

Table S2. Comparison of the demographic and medical data of ulcerative colitis patients who developed cancer (18 patients with 20 cancers) with ulcerative colitis patients who did not develop cancer (n = 232). * $p < 0.05$.

	**UC (232)**	**UC with cancer (20/18)**	**p**
Age at diagnosis of IBD, median [range], y	32 (5-76)	30 (13-73)	0.4
IBD duration at cancer Diagnosis, median [range], y	15 (5-52)	15 (1-38)	0.9
**Sex, No. (%)**			
females	112 (49%)	11 (61%)	0.29
males	120 (51%)	7 (39%)	
**Smoking habits (%)**			
current	17 (8%)	2 (11%)	0.6
never	145 (62%)	10 (56%)	0.39
former smoker	23 (10%)	2 (11%)	0.9
not specified	46 (20%)	4 (22%)	0.8
**Alcohol intake (%)**			
current	9 (4%)	0	-
never	171 (73%)	12 (100%)	0.5
former	2 (1%)	0	-
not specified	56 (22%)	0	-
**Montreal Classification**			
	[b]E1 34(13%)	[b]E1 0 (0%)	-
	E2 112 (48%)	E2 9 (50%)	0.8
	E3 78 (35%)	E3 9 (50%)	0.15
	-		
	-		

	-		
**Disease activity**			
remission	[e] 51(24%)	[e]9 (50%)	**0.0073***
mild	133 (56%)	7 (39%)	0.12
moderate	48 (20%)	2 (11%)	0.3
severe	0	0	-
**Extra-intestinal manifestations**	83 (35%)	3	0.08
**IBD-related surgery, No. (%)**	15 (6%)	0	-
Medication			
5- Aminosalicylate	202 (93%)	15 (83%)	0.6
Glucocorticoids	201 (87%)	0	-
IM (AZA/6-MP/MTX)	125 (57%)	5 (27%)	0.2
anti-TNFα	129 (56%)	2 (11%)	**0.00027***
Vedolizumab	44 (19%)	1 (5%)	0.15
Ustekinumab	15 (6%)	0	-
Combination therapy			
IM+anti-TNFα	9 (4.7)	2 (11%)	0.14
IM+ Ustekinumab	10 (4.3%)	0	-
IM+Vedolizumab	15 (6.4%)	0	-
